# Effect of High-Frequency Oscillatory Ventilation Combined With Pulmonary Surfactant in the Treatment of Acute Respiratory Distress Syndrome After Cardiac Surgery: A Prospective Randomised Controlled Trial

**DOI:** 10.3389/fcvm.2021.675213

**Published:** 2021-07-22

**Authors:** Yi-Rong Zheng, Yu-Qing Lei, Jian-Feng Liu, Hong-Lin Wu, Ning Xu, Shu-Ting Huang, Hua Cao, Qiang Chen

**Affiliations:** ^1^Department of Cardiac Surgery, Fujian Branch of Shanghai Children's Medical Center, Fuzhou, China; ^2^Fujian Children's Hospital, Fuzhou, China; ^3^Fujian Maternity and Child Health Hospital, Affiliated Hospital of Fujian Medical University, Fuzhou, China; ^4^Fujian Key Laboratory of Women and Children's Critical Diseases Research, Fujian Maternity and Child Health Hospital, Fuzhou, China

**Keywords:** high-frequency oscillatory ventilation, pulmonary surfactant, acute respiratory distress syndrome, congenital heart surgery, postoperative care

## Abstract

**Background:** This study aimed to evaluate the effects of pulmonary surfactant (PS) combined with high-frequency oscillatory ventilation (HFOV) or conventional mechanical ventilation (CMV) in infants with acute respiratory distress syndrome (ARDS) after congenital cardiac surgery.

**Methods:** A total of 61 infants with ARDS were eligible and were randomised to the CMV + PS group (*n* = 30) or the HFOV + PS group (*n* = 31) between January 2020 and December 2020. The primary outcomes were the changes in arterial blood gas parameters. The duration of mechanical ventilation, length of hospitalisation and the incidence of complications were considered secondary outcomes.

**Results:** A total of 61 infants completed the study. In the HFOV + PS group, the blood gas analysis results were significantly improved (*P* < 0.05), while the duration of mechanical ventilation and length of hospitalisation were shorter than the CMV + PS group (*P* < 0.05). However, the incidence of complications was not different between the two groups (*P* > 0.05).

**Conclusions:** Compared with the CMV + PS group, the HFOV + PS group showed significantly improved ABG variables and had a shortened length of hospitalisation and mechanical ventilation in infants with ARDS after cardiac surgery.

**Clinical Trial Registration:** Chinese Clinical Trial Registry; Number: ChiCTR2000039457.

## Introduction

The continuous improvement of cardiopulmonary bypass technology has increased the safety of surgical correction of congenital heart disease (CHD). However, acute lung injury and acute respiratory distress syndrome (ARDS) often occur after cardiopulmonary bypass (CPB). It is reported that up to 20% of cardiac surgery patients will develop ARDS during the perioperative period, and the mortality rate is as high as 80% (1, 2). The systemic inflammatory response and lung ischaemia-reperfusion injury caused by CPB during cardiac surgery are related to ARDS (3, 4). The cornerstone of ALI/ARDS management is lung-protective mechanical ventilation with low tidal volumes (5). Among them, high-frequency oscillatory ventilation (HFOV) applies tidal volume less than or equal to the anatomical dead space for rapid gas exchange has been proven to have a protective effect on the lungs (6, 7). In addition, the application of exogenous pulmonary surfactant (PS) replacement therapy in neonatal respiratory distress syndrome (NRDS) has been popularised, and the curative effect has been determined. However, the effect of paediatric ARDS needs to be studied further (1, 8). HFOV + PS inhalation therapy has demonstrated good effects in the treatment of various respiratory diseases in newborns and infants, such as NRDS, congenital diaphragmatic hernia and meconium aspiration syndrome (9, 10). However, the research of HFOV + PS for treating secondary ARDS after cardiac surgery in infants remains limited. The purpose of this study is to evaluate the efficacy and safety of HFOV + PS vs. conventional mechanical ventilation (CMV) + PS in the treatment of ARDS after cardiac surgery.

## Materials and Methods

### Population and Study Criteria

This investigation was a single-centre, prospective randomised controlled study conducted in the cardiac intensive care unit (CICU) from January 2020 to December 2020 at a provincial hospital in China. The trial was approved by the ethics committee of Fujian Maternity and Child Health Hospital (NO. 2020YJ181) and adhered to the tenets of the Declaration of Helsinki (as revised in 2013). In addition, informed parental written consent from all subjects was obtained.

The inclusion criteria were as follows: 1. Infants with ARDS undergoing repair of atrial septal defect (ASD) or ventricular septal defect (VSD) in our CICU. 2. The patient's anatomical treatment was satisfactory, cardiac function recovered well-after the operation, and haemodynamics were stable. The exclusion criteria included haemodynamically significant residual lesions, pulmonary venous obstruction and parents' decision not to participate.

### Allocation

After documenting parental consent, all infants were assigned to either HFOV + PS or CMV + PS using a table of computer-generated random numbers and sealed opaque envelopes.

### Respiratory Management

#### PS

The PS (porcine surfactant, Curosurf®, Chiesi Pharmaceutical SpA, Parma, Italy) was used for intratracheal injection. The infants in both groups were given 100 mg/kg in one dose within 6 h after ARDS diagnosis. Before application, the airway secretions were aspirated, and circulation was maintained stable to correct the acid-base disorder. After the PS injection, pressurise and ventilate rapidly for 1 min, then connect to a ventilator to maintain HFOV/CMV ventilation.

#### Mechanical Ventilation: HFOV + PS Group

A high-frequency oscillating ventilator (SLE 5,000, SLE UK, Croyden, United Kingdom) was used for mechanical ventilation before PS application. The ventilator's initial parameters were as follows: A frequency of 8–12 Hz and an inspiratory expiratory ratio of 1:1 were used in each case; The mean airway pressure (MAP) is initially set at 10–15 cmH_2_O and then gradually increased with steps of 1 cmH_2_O every 2–3 min until the oxygenation no longer improved; Then, the MAP was decreased by 1–2 cmH_2_O every 2–3 min until TcSaO_2_ decreased, and then added 1–2 cmH_2_O on the basis of this MAP. The amplitude was initially set at 30–40 cm H_2_O. After 1 h of mechanical ventilation, the ideal lung inflation was examined by chest radiography, and the right diaphragm was generally kept at the level of the ninth rib. The parameter adjustment is determined according to the results of the dynamic monitoring of blood gas analysis. After continuous ventilation for at least 10 min, the PS was injected through the tracheal tube. The changes in oxygen saturation (SaO_2_), heart rate, respiration and blood pressure were closely monitored during the treatment. If the infant suffered from apnoea, SaO_2_ or the heart rate dropped, the injection was suspended and oxygen was rapidly pressurised until the stable state was restored. After PS was applied, mechanical ventilation was continued, and the ventilator parameters were readjusted according to the SaO2 results, blood gas analysis and chest radiograph. Infants were extubated when they achieved the following criteria: haemodynamic stability, MAP <8 cm H_2_O, FiO_2_ < 40% and weaned sedation. The infants were transferred to conventional ventilation before extubation. After weaning, nasal continuous positive pressure ventilation (NCPAP) was administered through a nasal interface.

#### CMV + PS Group

The Stephanie ventilator (Fritz Stephan GmbH, Gackenbach, Germany) was used for CMV. The initial parameters: FiO_2_: 30–50%, PIP: 18–25 cm H_2_O, PEEP: 4–6 cm H_2_O, I/E: 1–1.5:1, RR: 30–40 times/min. According to the results of SaO_2_, arterial blood gas (ABG) analysis and chest radiograph, the parameters of the ventilator were adjusted, and continuous positive pressure ventilation (NCPAP) was given through nasal congestion after weaning.

### Definitions and Data Collection

#### Outcome Measures

The ventilatory settings, ABG analysis, oxygenation index (OI), duration of mechanical ventilation and complications of ventilation were recorded during the study period. The primary outcome was the changes of ABG parameters, and the secondary outcome was the duration of mechanical ventilation and length of hospitalisation. The diagnosis of ARDS was established according to the medical history, clinical manifestations and chest radiograph results which defined in 2015 by the Paediatric Acute Lung Injury Consensus Conference Group (PALCCG) (1) and met the following conditions: (1) hypoxaemia with OI ≥ 4 or oxygenation saturation index (OSI) ≥ 5; (2) new radiological lung infiltrates; (3) occurred within 7 days of a known clinical insult and (4) could not be explained by cardiac failure or fluid overload. (5) other patients with chronic lung/heart diseases, such as cyanotic heart disease, chronic lung disease and left ventricular dysfunction, to be included as long as they fulfilled the criteria above. The acute deterioration in oxygenation and pulmonary infiltrates could not be explained by their pre-existing diseases. (6) excluded patients with perinatal related lung disease. For infants younger than 44 weeks included in the study, we referred to the Montreux definition of neonatal ARDS (11). The diagnosis of ventilator-associated pneumonia (VAP) was based on the criteria established by the Centres for Disease Control and Prevention, aided by chest radiographs, positive sputum cultures, transtracheal fluid, bronchial washings and clinical findings (12). A VAP diagnosis was considered when it was identified after 48 h of mechanical ventilation. We analysed the demographics and clinical characteristics, surgical and CPB techniques, radiology and laboratory test results, ABG analysis, short-term outcomes, such as VAP, sepsis, length of mechanical ventilation, length of ICU stay and in-hospital mortality. ABG analysis was evaluated 30 min after starting HFOV or administering PS using a blood gas analyser (ABLTM 700 radiometer, Copenhagen, Denmark) and repeated every 4–6 h or more often, as needed. We elected to use OI [(FiO_2_ × MAP × 100) ÷ PaO_2_] to define the severity of PARDS. The severity of ARDS was defined as follows: mild 4 ≤ OI <8, moderate 8 ≤ OI <16, severe OI ≥ 16 (1).

### Statistical Analysis

Based on the improvement of ABG in the pre-experiment, assuming the difference between the two independent populations was 15%, α = 0.05 and β = 0.2, the number of participants needed was 27 in each group. Assuming a 10% attrition rate, the total sample size was 60 (30 per group).

Data were analysed using SPSS software version 25.0 for Windows (IBM SPSS Inc., Chicago, IL, USA). Independent continuous variables are presented as the mean ± standard deviation and were analysed by *t*-tests. Counts and percentages describe the enumeration data. Means were compared using Student's *t*-test, and Fisher's exact test was used for categorical data. The Mann–Whitney *U*-test was applied for nonnormally distributed data. A two-sided *P*-value of <0.05 was regarded as statistically significant.

## Results

### Baseline Data

**Figure 1:**
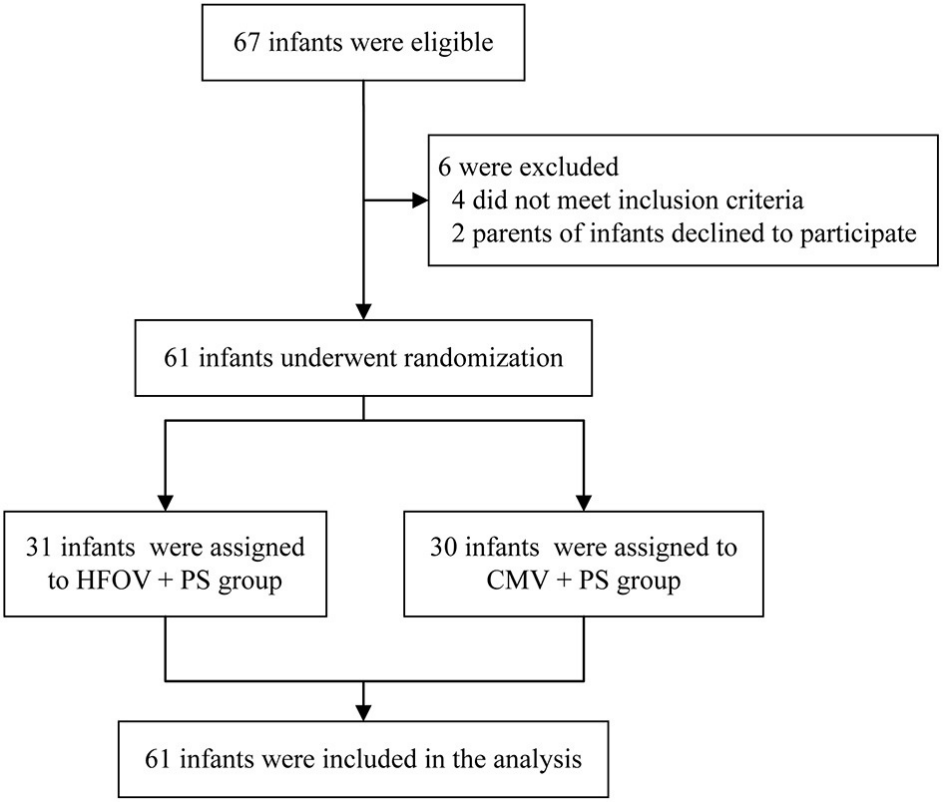
Participants' CONSORT flow diagram.

A total of 67 infants who underwent an ASD or VSD repair were screened between January 2020 and December 2020, of which four did not meet inclusion criteria, two parents of infants declined to participate. Ultimately, 61 infants were enrolled and finished the trial (31 in HFOV + PS group and 30 in CMV + PS group) (Figure 1). There were no significant differences between the two groups regarding their main clinical characteristics, including gender, GA, weight, the severity of ARDS or surgical data (Table 1). These results indicated that the two patient groups were homogeneous and comparable.

**Table 1:** Baseline characteristics^a^.

**Characteristics**	**HFOV+PS (*n* = 31)**	**CMV+PS (*n* = 30)**	***P*-Value**
Sex (Male/Female)	19/12	17/13	0.797
Gestation age at birth (weeks; mean ± SD)	38.2 ± 2.1	38.0 ± 2.2	0.658
Age at surgery (days; mean ± SD)	50.3 ± 15.7	46.7 ± 14.7	0.397
Weight at surgery (kg; mean ± SD)	4.3 ± 0.7	4.5 ± 0.8	0.406
Preoperative respiratory failure, *n* (%)	11 (35.5)	9 (30)	0.786
Duration of antibiotic administration (days; mean ± SD)	9.7 ± 0.8	8.9 ± 0.7	0.055
Pulmonary hypertension, *n* (%)	20 (64.5)	23 (76.7)	0.402
Duration of iNO (h; mean ± SD)	92.5 ± 31.2	101.3 ± 28.8	0.257
Operation time (h; mean ± SD)	3.8 ± 0.8	3.5 ± 1.1	0.319
CPB time (h; mean ± SD)	2.1 ± 0.3	2.0 ± 0.5	0.299
Severity of ARDS, *n*			
Mild	10	8	0.580
Moderate	17	20	
Severe	4	2	
Congenital heart disease, *n*			
VSD	18	21	0.426
ASD	13	9	

*iNO, inhaled nitric oxide; CPB, cardiopulmonary bypass; ARDS, acute respiratory distress syndrome; VSD, ventricular septal defect; ASD, atrial septal defect*.

a *Data reported as number and percentage, mean + standard deviation*.

### Primary Outcomes: ABG Analysis Results

**Figure 2:**
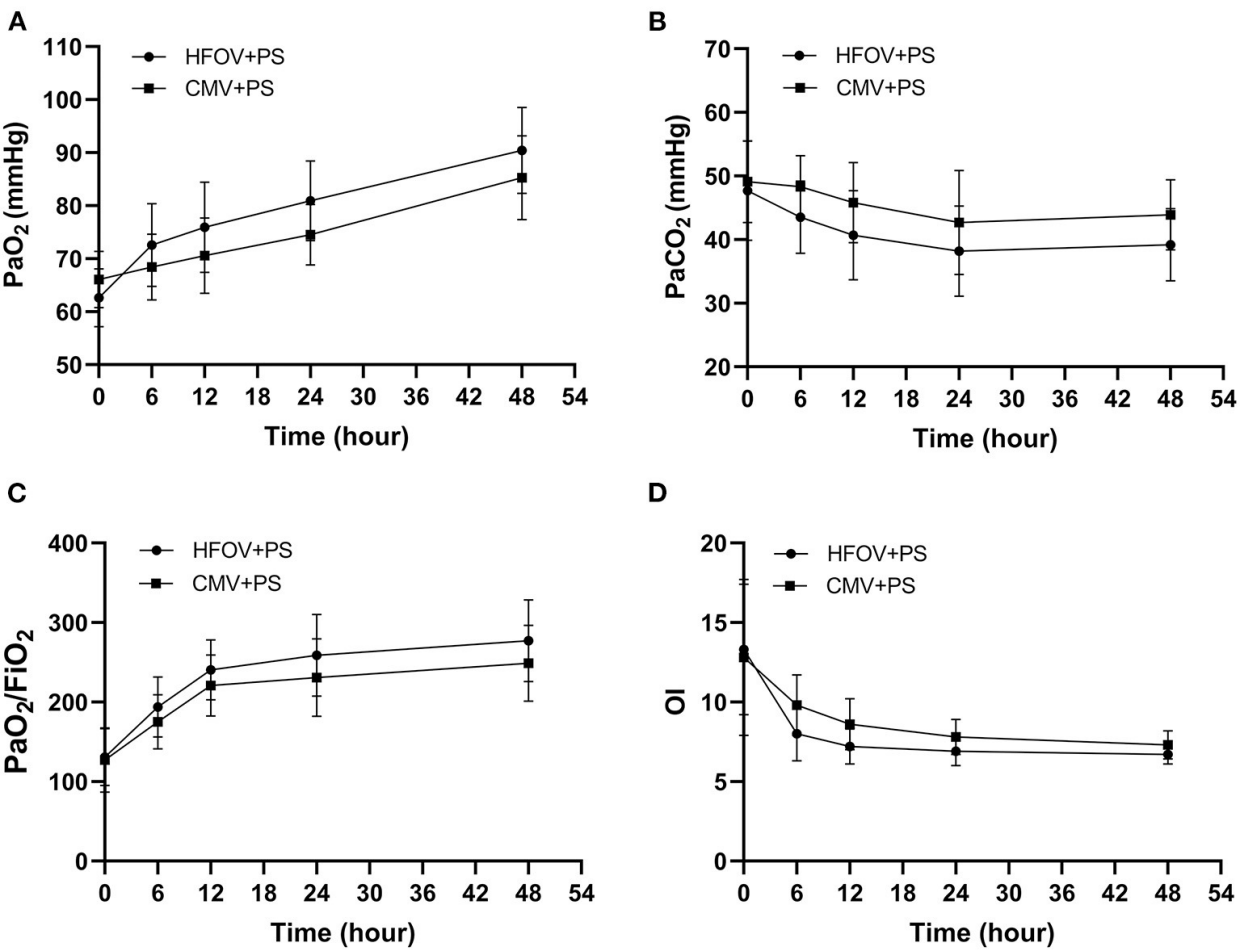
Arterial blood gas analysis results during the study period (compare the values at the beginning of the treatment and at 6, 12, 24, and 48 h, respectively). **(A)**: Evaluation of PaO_2_ in the two groups. The PaO_2_ in the HFOV + PS group was significantly higher than that in the CMV + PS group during the study period (*P* < 0.05). **(B)**: Evaluation of PaCO_2_ in the two groups during the study period. The PaCO_2_ in the HFOV + PS group was significantly lower than that in the CMV + PS group during the study period (*P* < 0.05). **(C)**: Evaluation of PaO_2_/FiO_2_ ratio in the two groups during the study period. The PaO_2_/FiO_2_ ratio in the HFOV + PS group was significantly lower than that in the CMV + PS group during the study period (*P* < 0.05). **(D)**: Evaluation of OI in the two groups during the study period. The OI in the HFOV + PS group was significantly lower than that in the CMV + PS group during the study period (*P* < 0.05).

There was no significant difference between the two groups of children at the beginning of treatment in arterial partial pressure of oxygen (PaO_2_), arterial partial pressure of carbon dioxide (PaCO_2_), PaO_2_/FiO_2_ and OI (*P* > 0.05). After 6, 12, 24, and 48 h of mechanical ventilation treatment, the blood gas analysis results were significantly improved and the difference was significant (*P* < 0.05) (Figure 2).

### Secondary Outcomes: Treatment Efficacy and Complications

During the application of HFOV + PS treatment, the heart rate decreased slightly (134 ± 17 vs. 143 ± 20 beats/min). In contrast, the mean arterial pressure (79 ± 14 vs. 83 ± 10 mmHg) and inotropic score (17.0 ± 5.5 vs. 16.0 ± 6.3) remained stable during this period, and the difference was significant. The length of hospital stays between the two groups of infants and the CICU length of stay in the HFOV + PS group were significantly shorter than in the CMV + PS group (*P* < 0.05). The duration of mechanical ventilation in the HFOV + PS group was also considerably shorter than that in the CMV + PS group (*P* = 0.03). There was no significant difference between the two groups in pneumothorax, VAP, sepsis, pulmonary haemorrhage and in-hospital mortality (*P* > 0.05) (Table 2).

**Table 2:** Vital signs and complications of the study infants^a^.

**Variable**	**HFOV+PS (*n* = 31)**	**CMV+PS (*n* = 30)**	***P*-Value**
Mean heart rate (beats/min, mean ± SD)	134 ± 17	143 ± 20	0.052
Mean arterial pressure, mean ± SD	79 ± 14	83 ± 10	0.110
Mean inotropic score (24 h postoperatively), mean ± SD	17.0 ± 5.5	16.0 ± 6.3	0.156
VAP, *n* (%)	4 (12.9)	2 (6.7)	0.671
Sepsis, *n* (%)	3 (9.7)	4 (13.3)	0.707
Pulmonary haemorrhage, *n* (%)	1 (3.2)	3 (10)	0.354
Pneumothorax, *n* (%)	0	2 (6.7)	0.492
In-hospital Mortality, *n* (%)	1 (3.2)	3 (10)	0.354
Postoperative duration of intubation (days; mean ± SD)	6.6 ± 2.8	8.7 ± 4.4	**0.030**
CICU length of stay (days; mean ± SD)	11.2 ± 4.5	14.1 ± 3.3	**0.006**
Total hospital length of stay (days; mean ± SD)	19.9 ± 2.9	21.7 ± 3.0	**0.032**

*VAP, ventilator-associated pneumonia; CICU, cardiac intensive care unit*.

a *Data reported as number and percentage, mean ± standard deviation*.

*The bold values in the table indicate that P value is less than 0.05, and the difference between the two groups is statistically significant*.

## Discussion

ARDS is a serious complication after cardiac surgery in children. The main manifestations are an increase in extensive alveolar permeability and refractory hypoxaemia. Hypoxia and acidosis damage pulmonary vascular endothelial cells and alveolar epithelial cells. Increased pulmonary microvascular permeability leads to alveolar and pulmonary interstitial oedema, damages alveolar type II cells and decreases endogenous PS production or release and activity. It also increases alveolar surface tension and decreases lung compliance leading to impaired lung function and decreased oxygenation levels (13, 14). Hypoxia and acidosis will affect haemodynamics in the short term, aggravate the degree of low cardiac output and even cause multiple organ failure. Mechanical ventilation is the essential treatment. However, studies have found that CMV can also cause and aggravate lung damage, mainly due to high airway pressure, high lung volume and repeated opening and closing of collapsed alveoli, which can trigger the release of inflammatory factors and secondary multiple organ dysfunction (15). Therefore, the lung-protective ventilation strategy has become the key to mechanical ventilation. The two most important aspects are limiting the excessive expansion of alveoli and applying relatively high PEEP to avoid the repeated closure of end-expiratory alveoli, thus reducing lung injury and promoting disease recovery. HFOV generates biphasic pressure changes with a tidal volume less than or equal to the anatomic dead space, low periodic pressure changes and hyperphysiological respiratory frequency oscillations. It then achieves an alveolar ventilation mode of effective gas exchange, which can uniformly expand the alveoli in a short time and improve gas exchange and lung compliance. HFOV is active in inhalation and exhalation during ventilation, effectively improving oxygenation and carbon dioxide emission without increasing barotrauma. Its hypoventilation strategy can prevent lung injury during mechanical ventilation and improve the survival rate (16, 17). Poddutoor et al. (18) conducted a study on 675 neonates receiving CMV. They found that 97 cases were switched to HFOV treatment after CMV treatment failed. In addition, the pulmonary oxygenation function and ABGs were significantly improved after 2 h. Our study also showed that the ABG results in the HFOV + PS group were significantly improved after treatment. Although the ABG results in the CMV + PS group were also improved, the effect was not as apparent as that in the HFOV + PS group. In addition, the average length of hospital stays and on-boarding time of infants in the HFOV + PS group were significantly shorter than those in the CMV + PS group. Also, the length of hospitalisation and the duration of mechanical ventilation in the HFOV + PS group were considerably shorter than those in the CMV + PS group. This was because, compared with CMV, the high-speed flow of gas produced by HFOV could quickly diffuse the PS to the surface of the alveoli, reduce its surface tension, further expand the alveoli, maintain their stability, improve the alveolar ventilation and ventilation function and further relieve the symptoms of hypoxia and acidosis.

PS replacement therapy can reduce alveolar surface tension, prevent alveolar collapse at the end of expiration, maintain functional residual capacity, stabilise alveolar pressure and reduce fluid leakage from the capillaries to the alveoli. At the same time, it can improve lung compliance and ventilation function and reduce ventilator parameters (19, 20). Studies have shown that early use of PS in children with ARDS can shorten the duration of mechanical ventilation and improve oxygenation (8, 21, 22). In addition, PS combined with mechanical ventilation can significantly increase PaO_2_/FiO_2_, decrease OI and significantly improve the oxygenation function of children with ARDS compared with simple mechanical ventilation (23, 24). This study also showed that the duration of mechanical ventilation and oxygenation of the HFOV + PS group were significantly lower than those of the CMV + PS group. The mechanism may be that HFOV can open occluded small airways and alveoli. The high-speed ventilation frequency and unique gas exchange mode accelerate the uniform distribution of the PS on alveolar walls. In addition, the immunomodulatory, biophysical and antibacterial properties of surfactants help to stabilise the alveoli and reduce alveolar-capillary oedema, ultimately improving lung function (24). When a PS is used in combination with HFOV, it can significantly improve breathing and reduce the ventilation duration.

Our study found that when HFOV was used in combination with the PS, the incidence of pulmonary haemorrhage, pneumothorax, VAP, intracranial haemorrhage and mortality in the two groups was not significant. Also, scholars have always been concerned about the safety of HFOV. Studies have found that the incidence of chronic lung disease and mortality after the application of HFOV is slightly superior to that of CMV, and there is no significant difference in the occurrence of complications, such as air leakage and brain injury (25, 26). Current clinical studies have shown that although exogenous PS can improve pulmonary gas exchange in infants with ARDS, its effect on reducing the mortality rate is uncertain (8, 27). Clinical research should be continued to determine which types of ARDS are effective for PS, and the appropriate timing, dosage and method of administration. Therefore, it is necessary to conduct a multicentre study to discuss related issues and provide evidence for the clinical use of exogenous PS in ARDS.

Although studies have demonstrated the effectiveness of surfactant and HFOV in neonates and infants in NRDS or ARDS. However, the application of HFOV combined with surfactant in ARDS during the perioperative period of congenital heart disease is still blank. This is the first study using HFOV+PS in infants with ARDS after congenital cardiac surgery. However, it has several limitations. First, this randomised controlled study was not a double-blind study; there were many subjective indicators in the research's evaluation criteria. Second, this was a single-centre study with a relatively small sample size. Finally, this study was limited to specific patients undergoing congenital cardiac surgery, and other patients might have different results. Therefore, additional, multicentre, prospective, randomised, double-blind, long-term studies with larger samples are needed.

## Conclusions

Compared with CMV + PS group, HFOV + PS can significantly improve the ABG variables and shorten the length of hospitalisation and mechanical ventilation in infants with ARDS after cardiac surgery. Meanwhile, it does not increase the incidence of complications.

## Data Availability Statement

The original contributions presented in the study are included in the article/Supplementary Material, further inquiries can be directed to the corresponding authors.

## Ethics Statement

The studies involving human participants were reviewed and approved by the ethics board of Fujian Medical University approved the study (NO. 2020KY039). Written informed consent to participate in this study was provided by the participants' legal guardian/next of kin.

## Author Contributions

QC and Y-RZ conceived the idea. H-LW and Y-QL conducted the analyses. NX and S-TH provided the data. All authors contributed to the article and approved the submitted version.

## Conflict of Interest

The authors declare that the research was conducted in the absence of any commercial or financial relationships that could be construed as a potential conflict of interest.
