# Supplementary material for: Effect of High-Frequency Oscillatory Ventilation Combined With Pulmonary Surfactant in the Treatment of Acute Respiratory Distress Syndrome After Cardiac Surgery: A Prospective Randomised Controlled Trial
Source: Front Cardiovasc Med. 2021 Jul 22;8:675213. doi: 10.3389/fcvm.2021.675213 (PMC8339213; doi:10.3389/fcvm.2021.675213)
Supplement: Supplementary file 1 [file Data_Sheet_1.PDF]

CONSORT 2010 checklist of information to include when reporting a randomised trial*

Section/Topic	Item No	Checklist item	Reported on Page Number/Line Number	Reported on Section/Paragraph
**Title and abstract**				
	1a	Identification as a randomised trial in the title	Page1/Line3	Title
	1b	Structured summary of trial design, methods, results, and conclusions (for specific guidance see Table 2)	Page2/Line25-41	Abstract/Para2-5
**Introduction**				
Background and objectives	2a	Scientific background and explanation of rationale	Page3/Line43-63	Introduction/Para1
	2b	Specific objectives or hypotheses	Page2/Line25-41	Introduction/Para1
**Methods**				
Trial design	3a	Description of trial design (such as parallel, factorial) including allocation ratio	Page4/Line67-72	Methods/Para1
	3b	Important changes to methods after trial commencement (such as eligibility criteria), with reasons		
Participants	4a	Eligibility criteria for participants	Page4/Line73-77	Methods/Para2
	4b	Settings and locations where the data were collected	Page4/Line67-72	Methods/Para1
Interventions	5	The interventions for each group with sufficient details to allow replication, including how and when they were actually administered	Page5-6/Line82-118	Methods/Para4-6
Outcomes	6a	Completely defined pre-specified primary and secondary outcome measures, including how and when they were assessed	Page6/Line120-146	Methods/Para7
	6b	Any changes to trial outcomes after the trial commenced, with reasons		
Sample size	7a	How sample size was determined	Page7/Line148-151	Methods/Para8
	7b	When applicable, explanation of any interim analyses and stopping guidelines		
Randomisation:				
Sequence generation	8a	Method used to generate the random allocation sequence	Page7-8/Line152-158	Methods/Para9
	8b	Type of randomisation; details of any restriction (such as blocking and block size)	Page7-8/Line152-158	Methods/Para9
Allocation concealment mechanism	9	Mechanism used to implement the random allocation sequence (such as sequentially numbered containers), describing any steps taken to conceal the sequence until interventions were assigned	Page3/Line78-81	Methods/Para2

Implementation	10	Who generated the random allocation sequence, who enrolled participants, and who assigned participants to interventions	Page3/Line78-81	Methods/Para2
Blinding	11a	If done, who was blinded after assignment to interventions (for example, participants, care providers, those assessing outcomes) and how		
	11b	If relevant, description of the similarity of interventions		
Statistical methods	12a	Statistical methods used to compare groups for primary and secondary outcomes	Page7-8/Line152-158	Methods/Para9
	12b	Methods for additional analyses, such as subgroup analyses and adjusted analyses	Page7-8/Line152-158	Methods/Para9
**Results**				
Participant flow (a diagram is strongly recommended)	13a	For each group, the numbers of participants who were randomly assigned, received intended treatment, and were analysed for the primary outcome	Figue1	Figue1
	13b	For each group, losses and exclusions after randomisation, together with reasons	Figue1	Figue1
Recruitment	14a	Dates defining the periods of recruitment and follow-up	Page8/Line161-167	Results/Para1
	14b	Why the trial ended or was stopped		
Baseline data	15	A table showing baseline demographic and clinical characteristics for each group	Table1	Table1
Numbers analysed	16	For each group, number of participants (denominator) included in each analysis and whether the analysis was by original assigned groups	Table1	Table1
Outcomes and estimation	17a	For each primary and secondary outcome, results for each group, and the estimated effect size and its precision (such as 95% confidence interval)	Page9/Line168-184	Results/Para2-3
	17b	For binary outcomes, presentation of both absolute and relative effect sizes is recommended	Page9/Line168-184	Results/Para2-3
Ancillary analyses	18	Results of any other analyses performed, including subgroup analyses and adjusted analyses, distinguishing pre-specified from exploratory	Page9/Line168-184	Results/Para2-3
Harms	19	All important harms or unintended effects in each group (for specific guidance see CONSORT for harms)		
**Discussion**				
Limitations	20	Trial limitations, addressing sources of potential bias, imprecision, and, if relevant, multiplicity of analyses	Page12-13/Line258-269	Discussion/Para5
Generalisability	21	Generalisability (external validity, applicability) of the trial findings	Page13/Line268-271	Conclusion/Para1
Interpretation	22	Interpretation consistent with results, balancing benefits and harms, and considering other relevant evidence	Page12/Line187-257	Discussion/Para1-4
**Other information**				
Registration	23	Registration number and name of trial registry		

Protocol	24	Where the full trial protocol can be accessed, if available		
Funding	25	Sources of funding and other support (such as supply of drugs), role of funders	Page13/Line276	

*We strongly recommend reading this statement in conjunction with the CONSORT 2010 Explanation and Elaboration for important clarifications on all the items. If relevant, we also recommend reading CONSORT extensions for cluster randomised trials, non-inferiority and equivalence trials, non-pharmacological treatments, herbal interventions, and pragmatic trials. Additional extensions are forthcoming: for those and for up to date references relevant to this checklist, see www.consort-statement.org.

Table 2 Items to include when reporting a randomized trial in a journal or conference abstract

**Item**	**Description**	**Reported on Page Number/Line Number**	**Reported on Section/Paragraph**
Title	Identification of the study as randomized	Page1/Line1-3	Title
Authors *	Contact details for the corresponding author	Page1/Line5-21	Title page/Para1-2
Trial design	Description of the trial design (e.g. parallel, cluster, non-inferiority)	Page1/Line1-3	Title
**Methods**			
Participants	Eligibility criteria for participants and the settings where the data were collected	Page4/Line67-77	Methods/Para1
Interventions	Interventions intended for each group	Page5-6/Line82-118	Methods/Para4-6
Objective	Specific objective or hypothesis	Page2/Line25-41	Introduction/Para1
Outcome	Clearly defined primary outcome for this report	Page6/Line120-146	Methods/Para7
Randomization	How participants were allocated to interventions	Page3/Line78-81	Methods/Para2
Blinding (masking)	Whether or not participants, care givers, and those assessing the outcomes were blinded to group assignment	/	/
**Results**			
Numbers randomized	Number of participants randomized to each group	Page8/Line161-167	Results/Para1
Recruitment	Trial status	/	/
Numbers analysed	Number of participants analysed in each group	Page8/Line161-167	Results/Para1
Outcome	For the primary outcome, a result for each group and the estimated effect size and its precision	Page9/Line168-184	Results/Para2-3
Harms	Important adverse events or side effects	/	/

Conclusions	General interpretation of the results	Page13/Line268-271	Conclusion/Para1
Trial registration	Registration number and name of trial register	/	/
Funding	Source of funding	Page13/Line276	

** this item is specific to conference abstracts*

From: Hopewell S, Clarke M, Moher D, et al. CONSORT for reporting randomized controlled trials in journal and conference abstracts: explanation and elaboration. PLoS Med. 2008;5(1):e20

rticle information: http://dx.doi.org/10.21037/tp-20-295.
*As the checklist was provided upon initial submission, the page number/line number reported may be changed due to copyediting and may not be referable in the published version. In this case, the section/paragraph may be used as an alternative reference.
